# Analysis of Bacterial Diversity in Different Types of Daqu and Fermented Grains From Danquan Distillery

**DOI:** 10.3389/fmicb.2022.883122

**Published:** 2022-07-04

**Authors:** Changhua Shang, Yujia Li, Jin Zhang, Shanling Gan

**Affiliations:** ^1^College of Life Sciences, Guangxi Normal University, Guilin, China; ^2^Key Laboratory of Ecology of Rare and Endangered Species and Environmental Protection (Guangxi Normal University), Ministry of Education, Guilin, China; ^3^Guangxi Key Laboratory of Landscape Resources Conservation and Sustainable Utilization in Lijiang River Basin (Guangxi Normal University), Guilin, China; ^4^School of Life Sciences, Sun Yat-sen University, Guangzhou, China

**Keywords:** bacterial diversity, Jiang-flavor Baijiu, Daqu, fermented grains, high-throughput sequencing

## Abstract

Bacterial communities in high-temperature Daqu and fermented grains are important for brewing Jiang-flavor Baijiu such as Danquan Baijiu. Daqu is a saccharifying and fermenting agent, which has a significant impact on the flavor of Baijiu. However, bacterial communities in three different types of samples from the Danquan distillery (dqjq_ck, dqjqcp, and dqjp3) were still unclear, which limited further development of Danquan Baijiu. “dqjq_ck” and “dqjqcp” indicate high-temperature Daqu at days 45 and 135, respectively. “dqjp3” indicates fermented grains. In this study, the bacterial communities of three samples were analyzed by Illumina Miseq high-throughput sequencing. The bacterial communities of three samples primarily composed of thermophilic bacteria and bacteria with stress resistance. The most abundant species in dqjq_ck, dqjqcp, and dqjp3 were *Comamonas, Bacillus*, and *unclassified Lactobacillales*, respectively. The main bacteria included *Bacillus, Comamonas, Myroides, Paenibacillus, Acetobacter, Kroppenstedtia, Staphylococcus, Saccharopolyspora, Planifilum, Lactobacillus, Acinetobacter, Oceanobacillus, Enterococcus, Thermoactinomyces, Lactococcus, Streptomyces, Saccharomonospora, Tepidimicrobium, Anaerosalibacter, unclassified_Lactobacillales, unclassified_Thermoactinomycetaceae_1, unclassified_Bacillaceae_2, unclassified_Bacillales, unclassified_Microbacteriaceae, unclassified_Rhodobacteraceae, unclassified_Actinopolysporineae*, and *unclassified_Flavobacteriaceae* in three samples (percentage was more than 1% in one of three samples). In our study, the succession of microbiota in three samples representing three important stages of Danquan Baijiu brewing was revealed. This article lays a good foundation for understanding the fermentation mechanism and screening some excellent indigenous bacteria to improve the quality of Danquan Baijiu in future.

## Introduction

Traditional Chinese Baijiu has a variety of flavor styles such as Jiang flavor, light flavor, and Nong flavor, and a long history (Jia et al., 2020). Jiang-flavor Baijiu is one of the most popular and typical flavor styles in traditional Chinese Baijiu, which is represented by the famous Maotai from Huairen city, Guizhou Province, and Danquan Baijiu from Hechi city, Guangxi Autonomous Region.

In the past decades, there were some important discoveries about the origin of Chinese Baijiu based on the study of historical documents and the important physical evidence for Baijiu brewing (i.e., distillers). Different dynasties were suggested about the origin of Chinese Baijiu such as the Eastern Han Dynasty, Tang Dynasty, Song Dynasty, and Yuan Dynasty (Fu, 2005; Feng, 2015). However, regardless of scholars' perspectives, there is a completely consistent consensus. Chinese distilled liquor entered the consumer market and rapidly developed in the Yuan Dynasty (Mao, 2014). Chinese Baijiu, brandy, whisky, vodka, rum, and gin are known as the six major distilled spirits in the world, which highlighted the importance of Chinese Baijiu (Zheng et al., 2017). In 2020 and 2021, the export volumes of Chinese Baijiu were 14,246 and 16,023 kiloliters (with $459.912 and $564.827 million of sales), respectively, which accounted for only 0.19 and 0.22% of the domestic Chinese Baijiu yield. In 2020 and 2021, the total sales of Chinese Baijiu were 583.6 and 603.3 billion. The huge sales show the importance of Baijiu in the economy and society. However, the very low export proportion of Chinese Baijiu indicates that there is much room for expansion in the foreign market.

The production of Danquan Baijiu utilizes traditional and spontaneous multi-strain (including bacteria and fungi) solid-state fermentation (Liu et al., 2019; Jiang et al., 2020). The quality and yield of Baijiu are closely associated with microbes during the fermentation process (Yang et al., 2018; Jiang et al., 2020). The correlation between the microbes producing Taorong-type Baijiu and the major volatile substances in the pit mud was analyzed, which revealed the importance of the related microbes (Liu et al., 2022). Li et al. explored the effect of brewing ingredients in pit muds on the yield of Luzhou-flavor Baijiu and the specific effect of pit mud microbes in different locations on Baijiu production (Li et al., 2022). Luzhou-flavor baijiu is yielded by the combined activities of various microbes, and its flavor is affected by specific microbial community structures and the expression of specific genes (Zhou et al., 2022). The aforementioned studies fully clarified the importance of microorganisms in the Baijiu fermentation process. Chinese Baijiu is one of the world's oldest distilled liquors, which typically originated from the use of Daqu fermentation starters. Daqu is a saccharifying and fermenting agent, which has a significant effect on the flavor of Chinese Baijiu (Zheng et al., 2011). The microbes in Danquan Baijiu brewing primarily originates from high-temperature Daqu (HTD) which is responsible for saccharification and fermentation processes (Yang et al., 2018; Li et al., 2019). For Danquan Baijiu, HTD is made from wheat and old Daqu, then subjected to shaping, fermenting, and ripening, which involves numerous microbes and enzymes (Wang et al., 2022). HTD significantly affects the yield and quality of Baijiu (Liu et al., 2019). With the higher HTD-making temperature, most yeasts and molds are destroyed, and the microbial community of HTD primarily propagates bacteria, leading to the microbial composition dominated by thermophilic bacteria (Gan et al., 2019; Xie et al., 2020). Moreover, as an important functional community in HTD, bacteria can produce various enzymes and plentiful Baijiu aroma substances or their precursors, which give Baijiu a unique flavor (Zhao et al., 2020). Therefore, systematic research on the diversity of bacterial community in HTD is necessary for fully understanding the bacterial resources and fermentation mechanism of Baijiu such as Danquan Baijiu and improving the quality and yield of Baijiu.

At present, high-throughput sequencing technology has been widely utilized for the analysis of microbial community of diverse environmental samples (Dreier et al., 2022). Compared with traditional fingerprint technology such as restriction fragment length polymorphism (RFLP), amplified fragment length polymorphism (AFLP), and random amplified polymorphic DNA (RAPD), high-throughput sequencing by Illumina MiSeq sequencing platform has many advantages such as high-throughput, fast rate, and accurate result (Hu et al., 2022). High-throughput sequencing has also been widely used to analyze the microbial community of various Daqu samples (Deng et al., 2020; Chen et al., 2021). Deng et al. investigated the differences of microbial communities and potential functions among four Jiang-flavor Daqu with different colors (named QW, QB, QY, and QR) by amplicon sequencing (Deng et al., 2020). Chen et al. used Illumina MiSeq sequencing to analyze the microbial community of special-flavor Baijiu Daqu in Jiangxi Province (Chen et al., 2021).

In this study, three samples (two HTDs and one fermented grains) were collected from the Danquan distillery. Through Illumina MiSeq high-throughput sequencing technology, the bacterial community in three samples was observed and compared. Then, the bacterial function was predicted by Phylogenetic Investigation of Communities by Reconstruction of Unobserved States (PICRUSTs) (Douglas et al., 2018). Therefore, this study is aimed to (a) fully reveal the bacterial community of three samples, (b) identify the most abundant species of three samples, and (c) understand the bacterial function in three samples.

## Materials and Methods

### Sample Collection

Totally, three samples (dqjq_ck, dqjqcp, and dqjp3) were collected from the Danquan distillery in Nandan county, Hechi city, Guangxi Autonomous Region. Among them, dqjq_ck and dqjqcp were high-temperature Daqu (HTD) bricks (as saccharification fermentation agent for liquor fermentation) fermented for 45 d and 135 d, and dqjp3 was fermented grains (i.e., residues from the brewing process); three parallel materials were mixed in equal quantities for eachsample (dqjq_ck, dqjqcp, and dqjp3).

The preparation process of Daqu was as follows. First, raw materials (wheats) were broken and then mixed with water and crushing mother Daqu according to a certain proportion (wheat:water:crushing mother Daqu=100%: 40%: 7%, weight ratio). Second, the mixture was filled into brick-shaped molds (38 ×28 × 6 cm), and the workers stepped on it to obtain sufficient cohesion and proper air permeability. Third, the Daqu bricks were transferred into fermentation chambers, then layer-by-layer stacked up with space between each two, and covered with straw to obtain a proper solid-state fermentation environment (temperature: 65–70°C, humidity: 80–90%) for microbes. After 45 d of fermentation, Daqu bricks named dqjq_ck was obtained. At last, the Daqu bricks dqjq_ck were transferred into another fermentation chamber (temperature: about 40°C, humidity: natural humidity), then layer-by-layer stacked up in the space between each two for 90 d of fermentation sequentially to obtain ripe Daqu bricks (dqjqcp). Then ripe Daqu bricks were used for the brewing process. Therefore, there were two important Daqu fermentation stages (a total of 135 d): 45 d of the first fermentation and 90 d of the second fermentation, which was the standard operation of Daqu preparation in the Danquan distillery. This is the reason for choosing 45 and 135 days for Daqu sampling.

### Metagenomic DNA Extraction

Metagenomic DNA was extracted from 0.5 g of each sample through an E.Z.N.A Mag-Bind Soil DNA Kit (Omega) in accordance with the manufacturer's instruction. DNA concentration was determined by using a Qubit 3.0 Fluorometer (Thermo Fisher Scientific). Qualified DNA was stored at −80°C until required.

### PCR Amplification and High-Throughput Sequencing

In this study, the V3-V4 region of 16S rDNA was amplified by sense primer 341F (CCTACGGGNGGCWGCAG) and antisense primer 805R (GACTACHVGGGTATCTAATCC). The PCR system was as follows: 15 μl 2×Hieff Robust PCR Master Mix, 1 μl 10 μM sense primer, 1 μl 10 μM antisense primer, 10 ng genomic DNA, and supplemented to 30 μl with ddH_2_O. The PCR condition was as follows: 94°C for 3 min, 5 cycles (94°C for 30 s, 45°C for 20 s, 65°C for 30 s), 20 cycles (94°C for 20 s, 55°C for 20 s, 72°C for 30 s), and 72°C for 5 min. Three DNA amplicons were sequenced by Miseq-Illumina sequencing platform in Sangon Biotech (China).

### Bioinformatics Analysis

Raw reads were processed by three kinds of software (cutadapt, PEAR, and PRINSEQ) to remove adaptors, unknown nucleotides, and low-quality reads. Then clean reads were obtained. The clean reads were clustered by Usearch based on a 97% similarity level to generate operational taxonomic units (OTUs). OTUs were performed homology comparison by the RDP classifier with RDP database, then annotated taxonomic positions at different taxonomic levels (phylum, class, order, family, and genus). Bacterial abundance and diversity were assessed in three samples based on α-diversity indexes. The sequencing depth was assessed by a sparse curve in order to check compliance with requirement of bioinformatics analysis. The similarity among bacterial composition of three samples was determined by UniFrac analysis. Principal coordinates analysis (PCoA) was performed by unweighted and weighted UniFrac. PICRUSt software was used to predict the functional potential of bacterial communities in three samples.

## Results

### Analysis of Bacterial Diversity in Three Different Samples

The Illumina Miseq platform in the three samples (dqjq_ck, dqjqcp, and dqjp3) generated a total 60,079, 66,533, and 48,559 raw reads, with average lengths of 460 bp, 459 bp, and 460 bp, respectively. There were 58,783, 64,669, and 48,130 clean reads in the three samples (dqjq_ck, dqjqcp, and dqjp3), with average lengths of 426, 426, and 423 bp, respectively. According to a 97% similarity level, the clean reads were divided, and a total 266 OTUs were obtained in the three samples. The proportion of each taxonomic position is shown in Supplementary Table 1. Gene copy numbers of bacterial communities are shown in Supplementary Table 2 for three samples.

**Figure 1:**
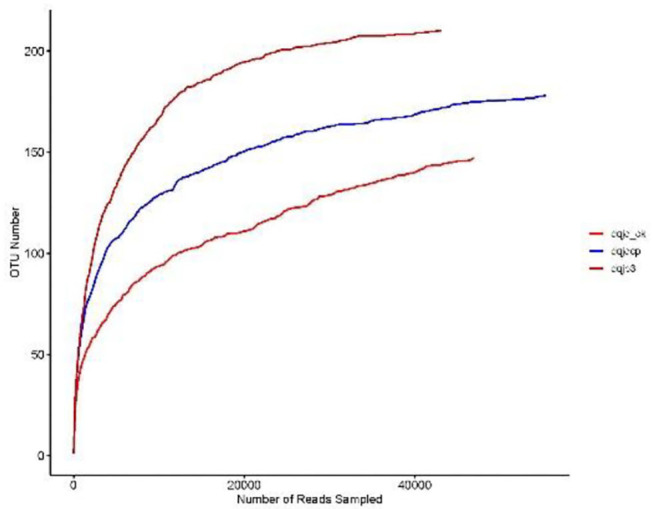
Sparse curves of three samples.

The sparse curve was used to evaluate the sequencing depth. From Figure 1, sparse curves were similar between the three samples, and the OTU number increased with the sequencing depth. With the gradual increase in sequencing depth, the OTU number of the three samples had an upward trend, which signified that the current sequencing depth was reasonable and sufficient to exhibit bacterial diversity in all samples. The α-diversity indexes in the three samples are shown in Table 1. The analysis of α-diversity showed that the highest and lowest richness (Chao index) were observed in dqjp3 and dqjq_ck, and the highest and lowest diversity (Shannon and Shannon even indexes) were observed in dqjqcp and dqjp3 (Table 1). The diversity indexes shown in Table 1 (including Shannon, Chao, Ace, Simpson, and Shannon even values) were utilized to evaluate the abundance and uniformity of the bacterial community. The richness estimator (Chao) was consistent with the number of OTUs. The bacterial OTUs and Chao were maximum in sample dqjp3, followed by sample dqjqcp. The Shannon index and Simpson index showed species diversity. The higher the Shannon index, the higher the community diversity. The bacterial Shannon value was the highest in sample dqjqcp and the lowest in sample dqjp3. The bacterial OTUs and Chao values of sample dqjp3 were the highest, while the Simpson value was the lowest.

**Table 1:** α-Diversity indexes in three samples.

**Sample**	**OTUs**	**Shannon**	**Chao**	**Ace**	**Simpson**	**Shannoneven**	**Coverage**
dqjp3	210	2.348468	217.0909	213.8911	0.215275	0.439203	0.999698
dqjq_ck	147	2.390457	199	219.5649	0.166389	0.479008	0.999148
dqjqcp	178	2.668424	201.2143	195.892	0.153707	0.514963	0.99953

### Analysis of Bacterial Community Structure in All Samples

In this study, phylum or genus (relative abundance>1.00% in one sample) was considered a dominant phylum or genus; other phyla or genera (relative abundance<1.00%) were classified as others. Sequences which were not be identified were classified as unclassified.

**Figure 2:**
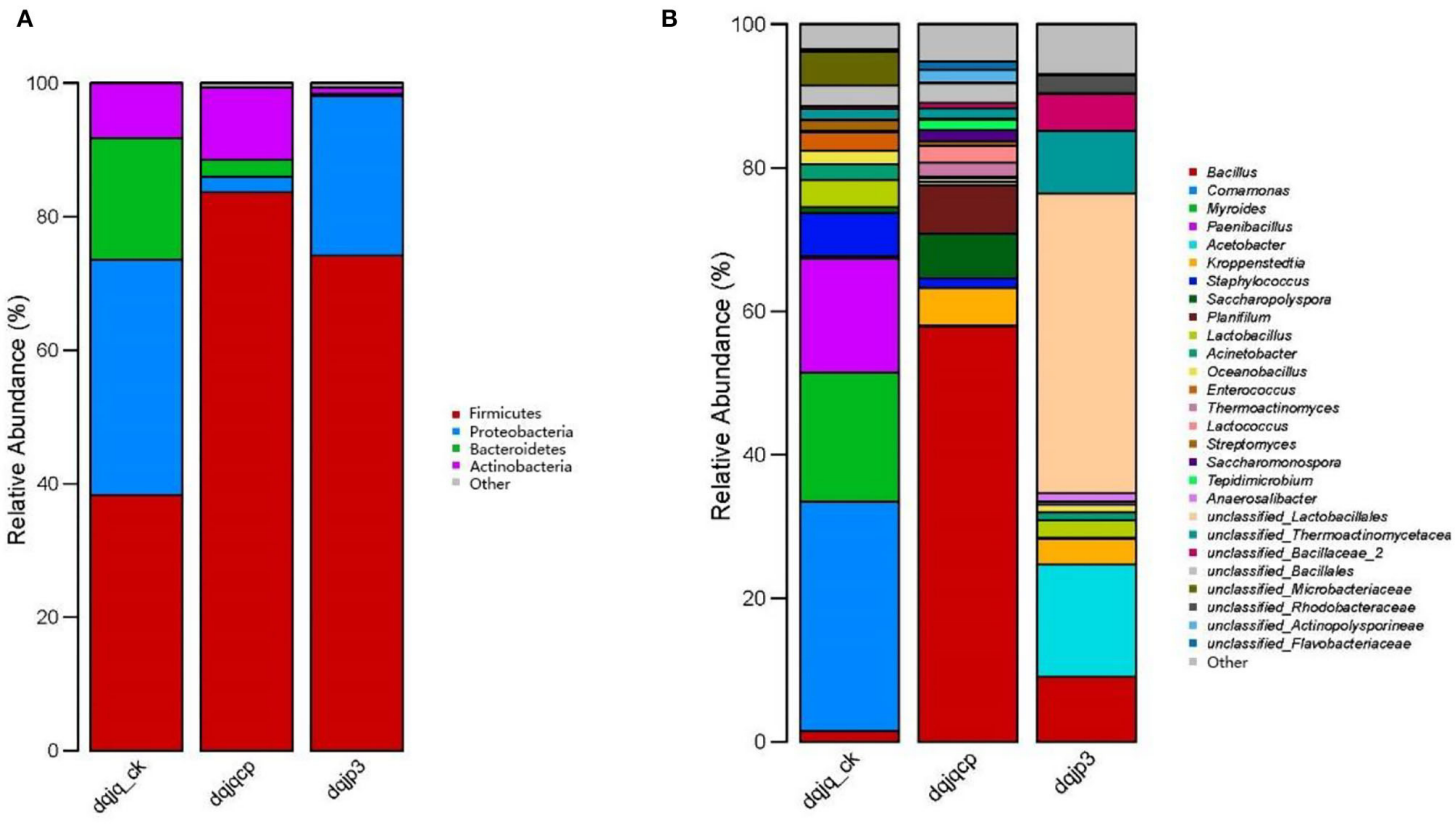
Taxonomic classification of bacterial reads retrieved from three samples at phylum and genus levels using RDP classifier. **(A)** Phylum level. **(B)** Genus level.

A total of 16 bacterial phyla were obtained from all samples, and four phyla were dominant among them, including Firmicutes, Actinobacteria, Proteobacteria, and Bacteroidetes, which accounted for most of total sequences (Figure 2A). Firmicutes accounted for 38.26, 83.66, and 74.18% in dqjq_ck, dqjqcp, and dqjp3, respectively, which was an absolute dominant phylum. Proteobacteria were also dominant in the three samples, although its abundance in sample dqjqcp was relatively low (2.31%). At the genus level, a total of 167 bacterial genera were obtained from all samples, and 27 genera were dominant among them. Genera with a higher abundance included *Bacillus, Comamonas, Myroides, Paenibacillus, Acetobacter*, and *unclassified_Lactobacillales* (Figure 2B). The main bacteria included *Bacillus, Comamonas, Myroides, Paenibacillus, Acetobacter, Kroppenstedtia, Staphylococcus, Saccharopolyspora, Planifilum, Lactobacillus, Acinetobacter, Oceanobacillus, Enterococcus, Thermoactinomyces, Lactococcus, Streptomyces, Saccharomonospora, Tepidimicrobium, Anaerosalibacter, unclassified_Lactobacillales, unclassified_Thermoactinomycetaceae_1, unclassified_Bacillaceae_2, unclassified_Bacillales, unclassified_Microbacteriaceae, unclassified_Rhodobacteraceae, unclassified_Actinopolysporineae*, and *unclassified_Flavobacteriaceae* in the three samples (percentage was more than 1% in one of three samples). Deng et al. found that *Kroppenstedtia* and *Bacillus* were predominant in all Daqu samples with different colors (named QW, QB, QY, and QR). *Saccharopolyspora* was predominant in QB and QR **(Deng et al.**, **2020****)**. The predominant bacterial communities included Bacillales, Lactobacillales, and Rhodospirillales. *Acetobacter* was the predominant bacterial genus in the surface part of Daqu, whereas *Kroppenstedtia, Oceanobacillus*, and *Bacillus* were the predominant bacterial genera in the central part of Daqu (Chen et al., 2021). In our study, the predominant genera also included the aforementioned genera in the studies of Deng et al. and Chen et al. However, the relative abundance of each predominant genus varied widely between our study and these two studies.

**Figure 3:**
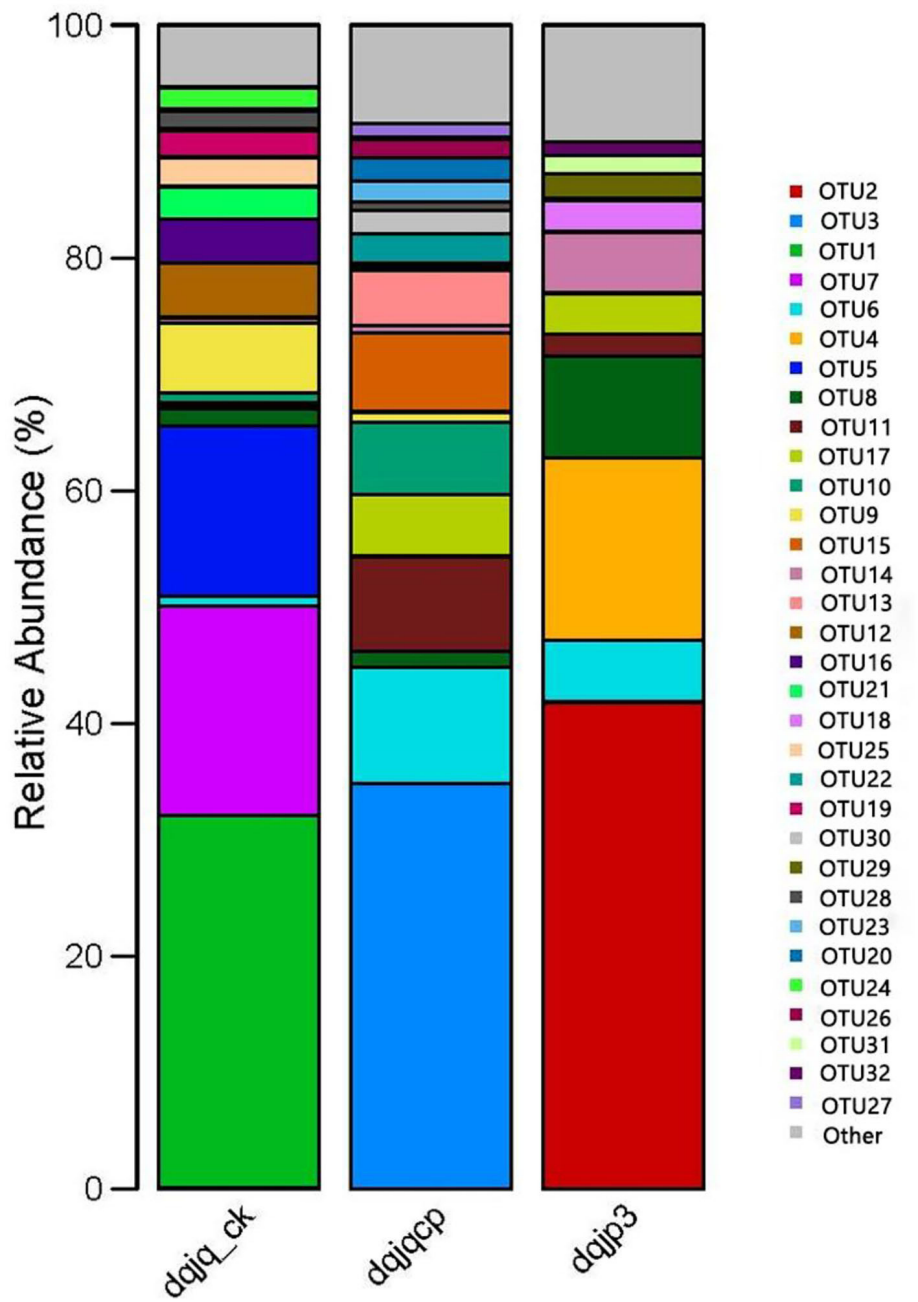
Bacterial community in three samples at the OTU level.

The bacterial community in the three samples was further characterized at the OTU level (Figure 3). In 266 OTUs found in all samples, there was one core OTU8, which was present in all samples with relative abundance (>1.00%). OTUs with higher abundance included OTU2, OTU3, OTU1, OTU7, OTU6, OTU4, OTU5, OTU8, OTU11, OTU17, OTU10, OTU9, OTU15, and OTU14.

The main genera in our three samples were described as follows. *Myroides* sp. ZB35 was used to produce aroma volatiles (22 esters) by solid-state fermentation on a rice medium **(Xiao et al.**, **2017****)**. At 192 h, the total concentration of the esters reached 1774 μg/kg. In addition, six novel esterase genes were found in *Myroides* sp. ZB35. Aroma volatiles containing 2-methylbutyl isovalerate, isoamyl isovalerate, isoamyl 2-methylbutanoate, and 2-methylbutyl 2-methylbutanoate were identified from *Myroides* sp. ZB35 extracts (Xiao et al., 2014). Many species of *Paenibacillus* genus are well-known plant growth promoters, which can promote plant nutrient uptake, control phytopathogens, and produce phytohormones. In addition to agricultural applications, *Paenibacillus* can produce a diverse variety of antimicrobials, enzymes, and exopolysaccharides for bioremediation (Grady et al., 2016). *Kroppenstedtia* and *Oceanobacillus* can secret amylase and lipase, and metabolize acids, which can enhance the use of sorghum and raw materials in Daqu (Yu et al., 2014; Wang X. et al., 2018). Differences in the production of fermented sausage flavor were found among different strains of *Staphylococcus xylosus* and *Staphylococcus carnosus* in meat simulation medium (Ravyts et al., 2010). *Staphylococcus gallinarum* and *Staphylococcus saprophyticus* were found in southern Daqu and were absent in northern Daqu (Zheng et al., 2015). The representative dominant microbes in low-temperature Daqu Qingcha, such as *Saccharopolyspora* sp., showed a significant positive correlation with the starch content and a significant negative correlation with saccharifying power (Hou et al., 2022). *Saccharopolyspora* sp. is necessary for the generation of the flavor substance in Maotai Baijiu production, so it might also be a desirable genus in Jiang-flavor Danquan Baijiu production (Gan et al., 2019). *Planifilum* belongs to Thermoactinomycetaceae family, which comprises thermophilic organisms with *Actinomyces*-like mycelial growth (Logan and Halket, 2011). Planifilum can secrete xylanase to degrade xylan and fix nitrogen (Bjarnadóttir et al., 2018). *Lactobacillus* is frequently detected during the brewing process of different types of Baijiu in China, which plays a necessary role in yielding aroma components (He et al., 2019). *Acinetobacter* and *Thermoactinomyces* showed the strongest positive correlation with acetic acid synthesis; these two types of bacteria positively correlated with the synthesis of more than two organic acids (Zhao et al., 2020). As a lactic acid bacterium, *Enterococcus* can be isolated during the fermentation of HTD from Maotai liquor, and *Enterococcus* was also present in fermented grains (Wang et al., 2015). *Lactococcus* presented the strongest positive correlation with lactic acid synthesis (Zhao et al., 2020). Qiu et al. found that although *Lactococcus garvieae* LD3 had high arginine utilization in the production of medium temperature Daqu liquor, the strain could accumulate more citrulline to increase the ethyl carbamate content in fermented grains (Qiu et al., 2016). Previous studies have demonstrated that *Streptomyces* spp. in Daqu can introduce geosmin into the brewing process, resulting in the earthy-off flavor of the liquor (Du and Xu, 2012; Du et al., 2013). *Saccharomonospora* could hydrolyze phenolic compounds into a non-toxic form and reduce phytotoxicity (Shivlata and Satyanarayana, 2015). *Tepidimicrobium* belongs to Clostridiales, which played an important role in cellulose degradation (Koeck et al., 2013; Slobodkin et al., 2020). *Anaerosalibacter* is a halotolerant bacterium, which is associated with sludge and animal matter (Rezgui et al., 2012; Dione et al., 2016).

### Differential Analysis of Bacterial Community in Three Samples

**Figure 4:**
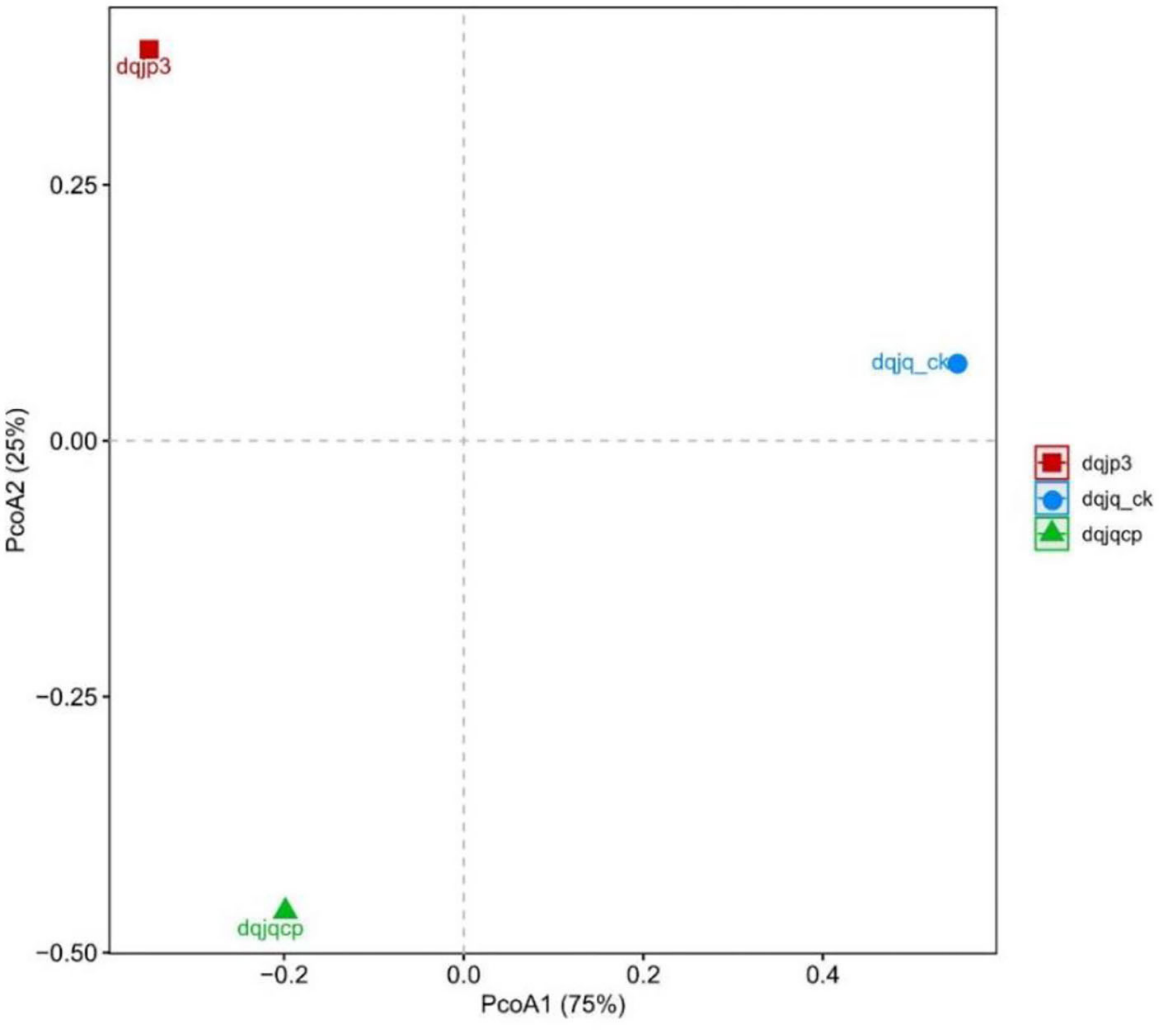
PCoA plot based on the 16S rDNA sequences from three samples.

Principal coordinates analysis was used to visualize the difference of bacterial community in the three samples (Figure 4). PCoA based on the Bray–Curtis distance showed that three different samples have obvious difference in spatial arrangement, which was probably due to the large differences in fermentation time and type. It was worth noting that the spatial distance between dqjq_ck and dqjp3 was farthest, suggesting that dqjq_ck and dqjp3 differ more in bacterial community.

PCoA showed significant differences among the bacterial communities of the three samples from the Danquan distillery (Figure 4). Although there were many identical genera in each sample, significant differences were apparent in the bacterial abundance among the three samples. In sample dqjq_ck, five genera such as *Comamonas* (32.00%), *Myroides* (18.03), *Paenibacillus* (15.92%), *Staphylococcus* (6.00%), and *unclassified_Microbacteriaceae* (4.70%) with high abundance accounted for 76.65% of the total abundance. In sample dqjqcp, five genera such as *Bacillus* (57.88%), *Planifilum* (6.76%), *Saccharopolyspora* (6.19), *Kroppenstedtia* (5.30%), and *unclassified_Bacillales* (2.78%) with high abundance accounted for 78.91% of the total abundance. In sample dqjp3, five genera such as *unclassified_Lactobacillales* (41.74%), *Acetobacter* (15.67%), *Bacillus* (9.04%), *unclassified_Thermoactinomycetaceae_1* (8.72%), and *unclassified_ Bacillaceae_2* (5.15%) with high abundance accounted for 80.32% of the total abundance. The significant differences of the bacterial communities occurred among the three samples, representing three important stages of Danquan Baijiu brewing, which were closely related to the environmental factors such as temperature, humidity, O_2_ content, and pH. Understanding the bacterial community succession in different stages of Baijiu production will help deepen the understanding of Baijiu fermentation mechanism and screen bacteria with excellent performance to prepare fortified Daqu for improving the quality of Baijiu. Some *Bacillus* strains with high yield of ethanol or flavor substance tetramethylpyrazine (TTMP) have been isolated and purified from three samples (dqjq_ck, dqjqcp, and dqjp3) based on our present study, and further functional research studies about these strains are ongoing.

### Prediction of Bacterial Functional Potential

**Figure 5:**
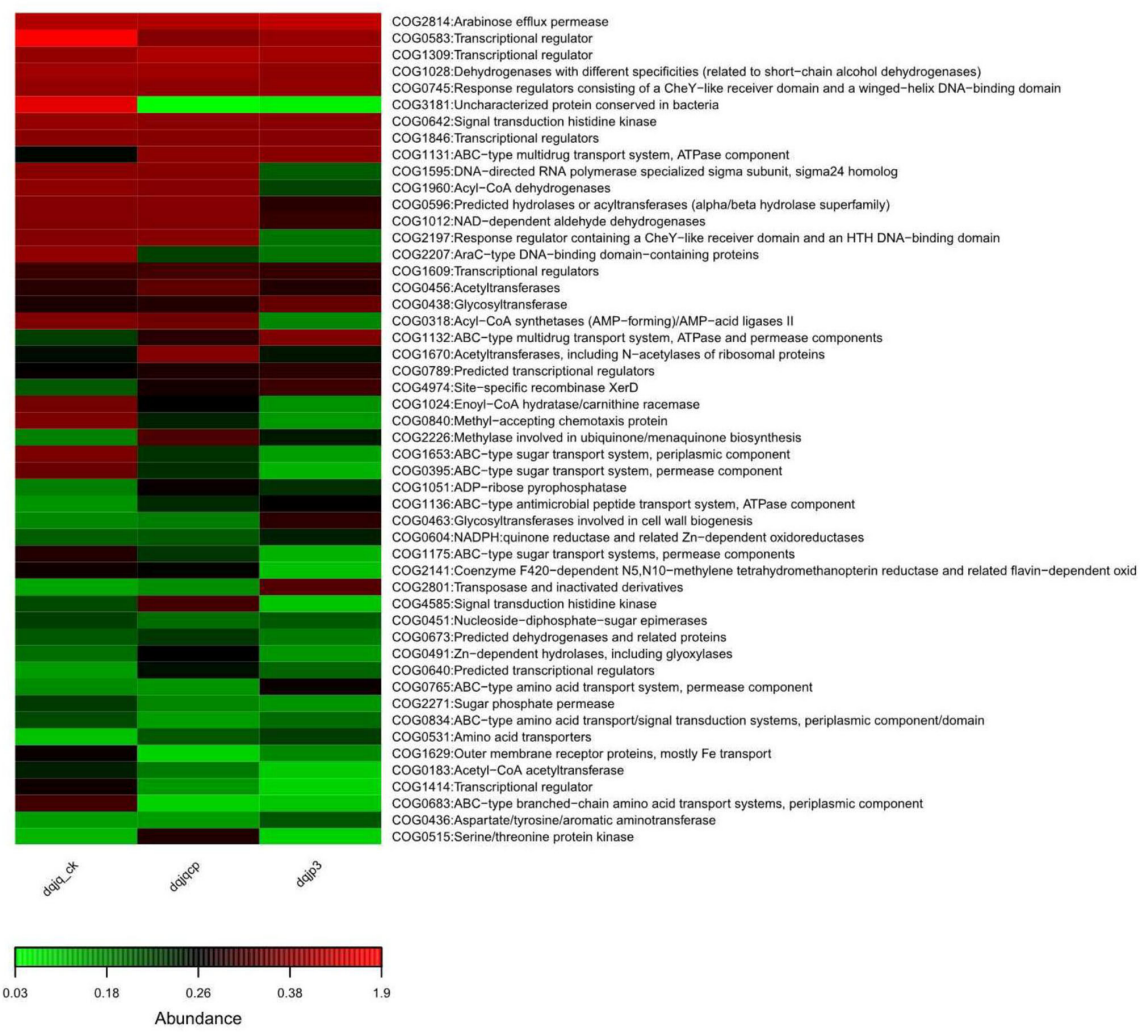
Overview of the bacterial COG profile in three samples.

To profoundly recognize the role of bacteria in the three samples, based on 16S rDNA data, PICRUSt was used to predict bacterial functional potential in the three samples. Further analysis was performed in view of COG database. More than 4,000 functional proteins belonging to 24 COG categories were identified to obtain a bacterial COG profile. Enrichment of metabolic functions in the three samples is shown in Figure 5, which indicated that the bacterial metabolism was intense. It can be observed from Figure 5 that categories G (carbohydrate transport and metabolism), K (transcription), and T (signal transduction mechanisms) dominated in all samples, and the relative abundance of category K was highest among all categories.

**Figure 6:**
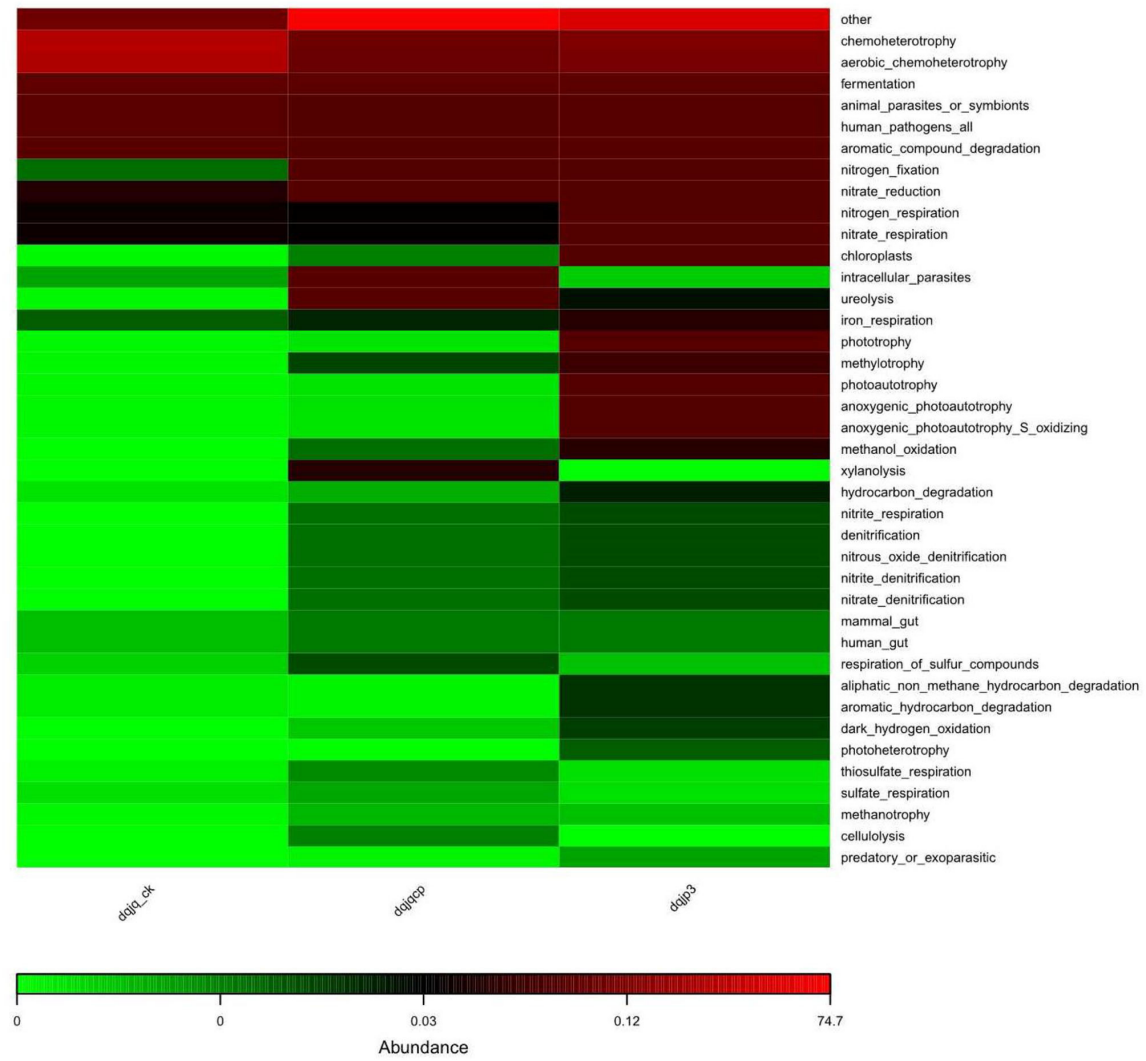
Prediction of bacterial functional potential.

The potential prediction of phenotypic functions in the three samples showed six dominant potential microbial phenotypes in all samples including chemoheterotrophy, aerobic chemoheterotrophy, fermentation, animal parasites or symbionts, human pathogens, and aromatic compound degradation (Figure 6).

## Discussion

High-temperature Daqu is manufactured by traditional spontaneous solid-state fermentation in the open environment (Huang et al., 2017). The polymicrobial co-fermentation process under the high-temperature condition is a distinct character of HTD, which is obviously different from other types of Daqu such as low-temperature Daqu for light-flavor Baijiu (Cai et al., 2021). Bacteria are important microbes, which produce a variety of aroma substances and enzymes in HTD.

In this study, Illumina MiSeq high-throughput sequencing was used to examine bacterial diversity of two HTDs (dqjq_ck and dqjqcp) and one fermented grain (dqjp3), respectively. Figure 2B shows that the dominant genera in the three samples were *Bacillus, Comamonas, Myroides, Paenibacillus, Acetobacter*, and *unclassified_Lactobacillales*. Different from our results, Jin et al. (2019) revealed dominant bacterial genera in HTD from Moatai and Xijiu towns, Guizhou province, China, were Bacillales, Enterobacteriales, and Lactobacillales, and Wang Y. et al. (2020) found that dominant bacterial genera in HTD from a traditional Maotai-flavor liquor production factory in Xiangyang, Hubei province, China, were *Thermoactinomyces, Staphylococcus, Lentibacillus, Kroppenstedtia*, and *Saccharopolyspora* (Jin et al., 2019; Wang Y. et al., 2020). This phenomenon suggested that HTD from different regions had a distinct bacterial community due to different environmental conditions.

The result of Figure 2B indicated that the most abundant species in dqjq_ck was *Comamonas*, and its relative abundance was highest in sample dqjq_ck. As the denitrifying bacteria, *Comamonas* can use oxygen present in nitrate or nitrite to oxidize the carbon, which leads to the formation of nitrogen gas from harmful nitrate or nitrite for human health when there is no oxygen available (Chu et al., 2022). *Comamonas* could be used as the indole-degrading genus (Yang et al., 2022). The dominant microbe *Comamonas* sp. was linked to the yield of a unique PHA member poly(3-hydroxyisobutyrate) with an isobutyrate-fed enrichment culture (Vermeer et al., 2022). *Comamonas* played a dominant role in tetracycline hydrochloride degradation and power generation in a microbial fuel cell (Zhang et al., 2021). *Comamonas testosteroni* YAZ2 could be used for the cleanup of polychlorinated biphenyl water pollution (Hara et al., 2021). The laccase enzymatic traits and delignification procedures of rice straw by *Comamonas testosteroni* FJ17 were investigated (Wang et al., 2021). *Comamonas thiooxydans* CG-1 had the ability for biodegradation of polyethylene terephthalate (a general plastic with non-degradable property) (Huang et al., 2022). *Comamonas* genus can degrade the raw materials in fermented grains, provide carbon source for the growth of other microorganisms, and participate in the formation of organic acids (Li and Qiu, 2021). *Comamonas acidovorans* has the ability of inhibiting spoilage mold. The existence of these bacteria can reduce the side effects caused by the invasion of spoilage mold in the brewing system (Yu et al., 2016). In brief, genus *Comamonas* could degrade a variety of pollutants in liquor production process.

The result of Figure 2B indicated that the most abundant species in dqjqcp was *Bacillus*, and its relative abundance was highest in sample dqjqcp. Genus *Bacillus* had been previously thought as a most represented bacterium with importance in a variety of Daqu (Zhang et al., 2014; Zheng et al., 2014; Hu et al., 2017). Genus *Bacillus* had been demonstrated to be the major Jiang flavor-producing bacterium for the yield of Jiang-flavor Baijiu, which could produce a variety of degrading enzymes such as protease, cellulase, amylase, and glucoamylase. These enzymes could hydrolyze macromolecules (protein, starch, etc.) to produce many flavor compounds during the production of Jiang-flavor Baijiu (Cai et al., 2021; Wu et al., 2021). Specific species of genus *Bacillus* and its quantity obviously affect the quality of Daqu and liquor style (Fan et al., 2018). For example, previous studies indicated that *Bacillus subtilis* and *Bacillus sarcina* could produce a red pigment, which might play an important role in Baijiu production (Zhao et al., 2009; Wei et al., 2014; Deng et al., 2020). Flavor metabolism of *Saccharomyces cerevisiae* by mixed culture with *Bacillus licheniformis* was improved during Chinese Maotai-flavor Baijiu fermentation, and the content of 12 flavor compounds, containing fatty acids and their esters, terpene, and aromatic compounds, was improved (Meng et al., 2015). *Bacillus licheniformis* stains MT-6 and MT-15 could produce the volatile compounds including C4 compounds, volatile acids, pyrazines, and aromatic and phenolic compounds, which could improve aroma concentration during the fermentation of Moutai-flavor Baijiu (Zhang et al., 2013). *Bacillus cereus* could make flavor compound ethyl hexanoate during the production process of Baijiu (Zhao et al., 2017). *B. cereus* and *B. licheniformis* were the most abundant bacteria to produce α-amylase in Jiuqu (Wang Z. M. et al., 2020). As the flavor-producing strains, *Bacillus amyloliquefaciens* and *Bacillus subtilis* could secrete α-amylase to enhance the saccharification process of raw materials and improve the contents of alcohols, esters, and diverse organic acids (Li et al., 2014; Yao et al., 2015). The relative abundance of genus *Bacillus* was higher at sites rich in oxygen in Daqu (He et al., 2019). Therefore, the most abundant species in sample dqjqcp, which was distributed in a well-ventilated area with more oxygen, is genus *Bacillus*. However, sample dqjq_ck was covered with thick rice straw with the least oxygen concentration, so relative abundance of genus *Bacillus* was the lowest. In addition, *Bacillus* could adapt to various unfavorable conditions such as heat and acid owing to the formation of spores (Wang W. et al., 2018; Mirza et al., 2022). In addition, genus *Bacillus* was the dominant genus in wheat (Zheng et al., 2015). Therefore, wheat as one of raw materials of two HTD samples (dqjqcp and dqjq_ck) was an important source of genus *Bacillus* (Du et al., 2019).

The result of Figure 2B indicated that the most abundant species in dqjp3 was *unclassified Lactobacillales*, and its relative abundance was highest in sample dqjp3. Lactobacillales is in the order of lactic acid bacteria. Lactobacillales was the dominant bacterium in Daqu owing to its extraordinary ability to adapt to various temperature and moisture, which helped it survive in high-temperature environments (Zhang et al., 2014; Wang and Xu, 2015). Lactobacillales can produce organic acids which may affect the growth of other microbes. For example, acetic acid can inhibit the growth of microbes and affect the structure of microbial community during the process of high-temperature fermentation (Nie et al., 2013). Furthermore, most genera of lactic acid-producing bacteria such as Lactobacillales could produce antibiotics such as bacteriocin, tetracycline, erythromycin, aminoglycoside, and vancomycin (Wu et al., 2017; Anisimova and Yarullina, 2019; Campedelli et al., 2019; Todorov et al., 2019; Arellano et al., 2020; Colautti et al., 2022). Lactobacillales and *Acetobacter* were found to affect the biomarkers through producing organic acids and bacteriocins and advance the occurrence of microbial community succession (Ma et al., 2022). In summary, Lactobacillales played an important role in flavor formation, microorganism antagonism, and microbial community succession.

Danquan Baijiu production involved three stages: the first stage (45 d of the first Daqu fermentation), the second stage (90 d of the second Daqu fermentation for the mature Daqu), and the third stage (mixing the crushed mature Daqu with the steamed sorghum for Baijiu brewing). After the end of each stage, the samples were collected, which in turn were dqjq_ck, dqjqcp, and dqjp3. Numerous studies had shown that *Bacillus* genus had the potential to produce alcohol. *Bacillus subtilis* can survive in different substrates because of its metabolic diversity and robust systems for the yield and secretion of a diversity of enzymes (Stülke and Hillen, 2000; Zhang and Zhang, 2010; Gu et al., 2018; Banerjee et al., 2019). *Bacillus subtilis* with innate ability to ferment a diversity of carbohydrates from mono-, di-, oligo-, and polysaccharides (such as starch, xylan, galactan, pullulan, arabinan, rhamnogalacturonan, and pectin) seems promising for inexpensive consolidated bioprocessing of bioethanol production using renewable plant biomass and wastes (Maleki et al., 2021). In addition, the ethanologenic *Bacillus subtilis* AP was estimated without and with Xyn-2 for bioethanol production from wheat bran. The strain *Bacillus subtilis* AP could produce 5.5 g/L ethanol, with a yield of 22.6% in consolidated bioprocessing (Rajabi et al., 2022). The proportions of *Bacillus* genus in dqjq_ck, dqjqcp, and dqjp3 were 1.43, 57.88, and 9.04%, respectively. With the higher HTD-making temperature, most yeasts and molds are destroyed, and the microbial community of HTD primarily propagates thermophilic bacteria (Gan et al., 2019; Xie et al., 2020). Therefore, we inferred that as a kind of thermophilic bacteria, *Bacillus* genus might play an important role in the bioethanol production for Danquan Baijiu brewing. Through our study, the succession of microbiota in the three samples representing the three important stages of Danquan Baijiu brewing were revealed. Our results will provide an important theoretical basis for enhancing the brewing process and the quality of Jiang-flavor Danquan Baijiu.

The result of functional potential and microbiome phenotype prediction is shown in Figures 5, 6. The predicted COG functional profile by PICRUSt showed that four functional categories with higher relative abundance in the three samples included K (transcription), E (amino acid transport and metabolism), G (carbohydrate transport and metabolism), and C (energy production and conversion) among all functional categories. In the meantime, amino acid transport and metabolism also require energy. In HTD, amino acids took part in Maillard reaction at high temperature, which gave HTD unique Jiang flavor (Gan et al., 2019; Feng et al., 2020). The noticeable expression of the functional category G (carbohydrate transport and metabolism) could provide a lot of energy for material transport, the reproduction and growth, and respiration in microbes. Carbohydrate metabolism and amino acid metabolism are very vital for microbes, which play an important role in the survival of microbes and the implementation of related functions. The KEGG-based analysis indicated that Baijiu Daqu and huangjiu wheat Qu microbiota were enriched with genes related to carbohydrate metabolism, amino acid metabolism, and energy metabolism. This result suggested that Daqu and wheat Qu microbiota had huge potential for the degradation of raw materials (such as wheat, rice, and barley) and flavor compound metabolism, which was consistent with our study (Zhang et al., 2022). Cai et al. suggested that the dominant microbial functions (such as E, G, and C) were the most vital functions in low-temperature Daqu (LTD), which indicated that the fermentation of LTD depended on the transport and metabolism of carbohydrates and amino acids; meanwhile, the life activity of microbes relied on energy metabolism (Cai et al., 2021). Given all that, categories E, G, and C were vital for different types of Qu.

Simultaneously, all samples also had a higher relative abundance in categories K (transcription), J (translation, ribosomal structure, and biogenesis), L (replication, recombination, and repair), and M (cell wall/membrane/envelope biogenesis), which indicated that bacteria in all samples maintained the frequent metabolism and had a high ability of growth and reproduction. Furthermore, all samples also had a high relative abundance in categories P (inorganic ion transport and metabolism) and H (coenzyme transport and metabolism). The transport of different inorganic ions is essential for bacterial metabolism, which can be utilized as cofactors of a variety of enzymes to advance different biochemical reactions. About coenzyme transport and metabolism, enzymes in HTD could catalyze the metabolism of amino acids to enhance the fermentation rate and provide substrates for Maillard reaction under high-temperature conditions, which caused Jiang flavor of HTD [7, 8] (Gan et al., 2019; Xie et al., 2020).

Traditional fermented foods such as Baijiu are frequently produced by spontaneous fermentation with multiple microbes. Environmental factors play important roles in microbial succession. Models were constructed to predict the population of core microbiota by processing parameters (room humidity and room temperature) (Ban et al., 2022). This research will be helpful for regulating microbes *via* controlling processing parameters in spontaneous fermentation such as Daqu fermentation (Ban et al., 2022). The microbial community succession (MCS) was driven by environmental factors such as temperature, humidity, CO_2_, O_2_, acidity, and moisture during fermentation of Nongxiangxing Daqu, and each environmental factor drove the occurrence of MCS by different mechanisms (Ma et al., 2022). The primary driving factors of MCS were acidity, moisture, and temperature for fermentation of Nongxiangxing Daqu (Ma et al., 2022). In our study, the fermentation temperature and humidity of dqjq_ck and dqjqcp were 65–70°C/80–90% and about 40°C/natural humidity, respectively. We inferred that the significant differences in environmental factors such as temperature and humidity affected MCS in samples dqjq_ck and dqjqcp. Traditional spontaneous fermentation with MCS and the changes of environmental factors result in an unstable quality of fermented foods in the microecosystem (He et al., 2020). He et al. found that a higher abundance affected the yield of flavor metabolites and changed the interspecies interactions of microbial community (He et al., 2020). Then He et al. investigated the bioturbation effect of fortified Daqu inoculating *Bacillus* strains on microbial composition and flavor metabolite and found that fortified Daqu could reduce the yield of lactic acid (He et al., 2020). More studies had explained that the acidification of fermentation cellar was primarily derived from the accumulation of lactic acid bacteria. In our study, as lactic acid bacterium, *unclassified Lactobacillales* had the highest abundance in fermented grains (i.e., sample dqjp3), which perhaps affected the flavor and quality of Baijiu. The fortified Daqu could help improve the flavor and quality of Baijiu. The indigenous microorganisms can better adapt to the local environment. Therefore, we isolated the suitable microorganisms from fermented grains such as *Bacillus* strains to improve the flavor and quality of Danquan Baijiu.

## Conclusion

In summary, the bacterial communities of the three samples from the Danquan distillery were analyzed. This article discussed the dominant genera with the potential for the improvement of the quality of Danquan Baijiu in the three samples. The most abundant species in dqjq_ck, dqjqcp, and dqjp3 were *Comamonas, Bacillus*, and *unclassified Lactobacillales*, respectively. Genus *Comamonas* could degrade a variety of pollutants in the fermentation process. Genus *Bacillus* could adapt to various unfavorable conditions, which was the major Jiang flavor-producing bacterium for the yield of Jiang-flavor Baijiu. The *unclassified Lactobacillales* played an important role in flavor formation, microorganism antagonism, and microbial community succession. With the changes in environmental conditions (fermentation temperature and humidity) and sample types, the microflora also underwent the corresponding adaptive changes. The results would lay the foundation for the isolation of some excellent indigenous bacteria and the construction of their genetic engineering modification to prepare fortified Daqu and improve the quality of Danquan Baijiu in future.

## Data Availability Statement

The datasets presented in this study can be found in online repositories. The names of the repository/repositories and accession number(s) can be found below: NCBI BioSample - SAMN26292234, SAMN26292235, and SAMN26292236.

## Author Contributions

CS: conceptualization, writing-review and editing, supervision, project administration, funding acquisition, and methodology. YL: software. YL and JZ: validation. CS, YL, and JZ: formal analysis. YL, JZ, and SG: investigation and data curation and writing-original draft preparation. JZ and SG: visualization. All authors have read and agreed to the published version of the manuscript.

## Funding

This research was financially supported by Guangxi Key Research and Development Program (AB21220057 and 2021AB27009), Research Funds of the Guangxi Key Laboratory of Landscape Resources Conservation and Sustainable Utilization in Lijiang River Basin, Guangxi Normal University (LRCSU21Z0207), Research Funds of Key Laboratory of Ecology of Rare and Endangered Species and Environmental Protection (Guangxi Normal University), Ministry of Education, China (ERESEP2022Z11), National Training Program of Innovation and Entrepreneurship for Undergraduates (202210602064), and Innovation Project of Guangxi Graduate Education (YCSW2022178 and XJCY2022011).

## Conflict of Interest

The authors declare that the research was conducted in the absence of any commercial or financial relationships that could be construed as a potential conflict of interest.

## Publisher's Note

All claims expressed in this article are solely those of the authors and do not necessarily represent those of their affiliated organizations, or those of the publisher, the editors and the reviewers. Any product that may be evaluated in this article, or claim that may be made by its manufacturer, is not guaranteed or endorsed by the publisher.

## Supplementary Material

Supplementary Table 1 The proportion of OTUs in three samples.

Supplementary Table 2 Gene copy numbers of bacterial communities in three samples.
